# Epigenetic Functions of Smchd1 Repress Gene Clusters on the Inactive X Chromosome and on Autosomes

**DOI:** 10.1128/MCB.00145-13

**Published:** 2013-08

**Authors:** Anne-Valerie Gendrel, Y. Amy Tang, Masako Suzuki, Jonathan Godwin, Tatyana B. Nesterova, John M. Greally, Edith Heard, Neil Brockdorff

**Affiliations:** Department of Biochemistry, University of Oxford, Oxford, United Kingdoma; Institut Curie, Unité de Génétique et Biologie du Développement, Paris, Franceb; MRC Clinical Sciences Centre, Faculty of Medicine ICSTM, Hammersmith Hospital, London, United Kingdomc; EMBL-European Bioinformatics Institute, Wellcome Trust Genome Campus, Hinxton, Cambridge, United Kingdomd; Albert Einstein College of Medicine, Bronx, New York, USAe

## Abstract

The *Smchd1* gene encodes a large protein with homology to the SMC family of proteins involved in chromosome condensation and cohesion. Previous studies have found that Smchd1 has an important role in CpG island (CGI) methylation on the inactive X chromosome (Xi) and in stable silencing of some Xi genes. In this study, using genome-wide expression analysis, we showed that Smchd1 is required for the silencing of around 10% of the genes on Xi, apparently independent of CGI hypomethylation, and, moreover, that these genes nonrandomly occur in clusters. Additionally, we found that Smchd1 is required for CpG island methylation and silencing at a cluster of four imprinted genes in the Prader-Willi syndrome (PWS) locus on chromosome 7 and genes from the protocadherin-alpha and -beta clusters. All of the affected autosomal loci display developmentally regulated brain-specific methylation patterns which are lost in *Smchd1* homozygous mutants. We discuss the implications of these findings for understanding the function of Smchd1 in epigenetic regulation of gene expression.

## INTRODUCTION

Smchd1 (for structural maintenance of chromosomes hinge domain containing 1) is a noncanonical member of the SMC protein family, which is involved in chromosome condensation and cohesion, segregation, and DNA repair (1). An *N*-ethyl-*N*-nitrosourea (ENU) mutagenesis screen identified the *Smchd1* locus as a dominant modifier of position effect variegation in mammals (2, 3). It was further shown that female homozygotes display midgestation embryo lethality (at approximately embryonic day 11.5 [E11.5]), whereas male homozygotes survive to adulthood (3). Together these observations suggested a role for Smchd1 in X chromosome inactivation. In further studies it was found that Smchd1 is indeed concentrated on the inactive X chromosome (Xi) in interphase nuclei (2). Moreover, we recently showed that Smchd1 is important for DNA methylation of a large proportion of Xi CpG islands (CGIs) (4) and the long-term silencing of selected Xi genes (2), with the latter providing a likely explanation for homozygous mutant female embryo lethality. Early markers of X inactivation, including expression and localization of Xist RNA, and accumulation of Polycomb-mediated histone modifications are largely unaffected in *Smchd1* mutants (2).

The viability of male *Smchd1* homozygotes suggests that the primary function of Smchd1 is in X chromosome inactivation. However, Smchd1 is conserved in nonmammalian vertebrates and in higher plants, neither of which have an X inactivation system, thus suggesting that mammals have coopted Smchd1 into the X inactivation system and that the protein has a wider evolutionarily conserved function. Studies on Smchd1 in Arabidopsis have identified a role in RNA-dependent DNA methylation, although in this instance the relevant homologues encode truncated proteins, corresponding only to the hinge (5) or the ATPase domain (6). The latter was also shown to play a role in constitutive heterochromatin condensation and silencing of loci located in these regions (7). Interestingly, Lorkovic et al. also showed that the hinge and the ATPase domain proteins (called MORC) can form heterodimers, therefore reconstituting an Arabidopsis functional analogue of Smchd1 (6). Full-length Smchd1 homologues are also found in Arabidopsis and have been implicated in DNA repair pathways (8). Consistent with this, the human Smchd1 homologue was identified in a screen for factors that facilitate homologous recombination (9).

It is not yet clear how Smchd1 acts in maintaining CGI methylation and gene silencing on the inactive X and whether it could play a role in the process of heterochromatin compaction as a mechanism to maintain proper gene repression, similarly to the MORC ATPases in Arabidopsis. It is also not clear whether Smchd1 has a role in epigenetic regulation of autosomal genes in mammals. To address these questions, we have compared the patterns of gene expression on the X chromosome and on autosomes between wild-type and homozygous mutant (*Smchd1*^*−/−*^) female embryos. We find that 10% of X-linked genes are derepressed in mutant embryos and that these tend to be located in clusters. Those Xi genes that are derepressed in *Smchd1*^*−/−*^ embryos are not strongly affected in *Dnmt3b*^*−/−*^ female embryos where CGI methylation is lost, indicating that Smchd1 mediates silencing independent of its effect on CGI methylation. Additionally, we find that Smchd1 is required to repress a limited number of clustered autosomal loci in both male and female embryos. Interestingly, we find that the Prader-Willi syndrome (PWS) cluster that is subject to parent-of-origin specific imprinting (10) and the Protocadherin-alpha (*Pcdha*) and *-beta* (*Pcdhb*) gene clusters, the former of which is known to be monoallelically expressed in single neurons (11), are misregulated in mutant embryos. Misexpression of these clusters is linked to hypomethylation of associated CGIs, suggesting a direct parallel with Smchd1 function in X inactivation.

## MATERIALS AND METHODS

### Mouse strains and cell lines.

Animal studies were carried out under the United Kingdom Home Office ASPA project, licence numbers 70/6349 (until 2011) and 30/2800. The *Smchd1* mutant allele used in this study is the *MommeD1* null allele described in reference 2. Mice carrying the *Smchd1* mutant allele were maintained as heterozygotes in the FVB/N background, where the allele was originally isolated (3). Mice were genotyped by sequencing a PCR product overlapping exon 23, where the point mutation is located (2). *Smchd1* mutant embryos were obtained from timed matings of *Smchd1* heterozygote intercrosses, and detection of a vaginal plug was counted as embryonic day 0.5 (E0.5). *Dnmt3b* mutant embryos were obtained from crosses with conditional floxed alleles and the oocyte-specific CRE recombinase ZP3-CRE (12). Embryos were sexed and genotyped by PCR from extraembryonic ectoderm (at E6.5) or embryo fragments (at E9.5 and E10.5) as described previously (2, 13). For allele-specific expression analysis, *Smchd1*^*+/−*^ heterozygote mice were backcrossed twice to strain DBA/2J. F_2_ progeny were selected for heterozygosity at *Smchd1* and homozygosity for the DBA/2J single nucleotide polymorphisms (SNPs) previously identified. F_2_ males were crossed with FVB/N *Smchd1* heterozygote females, and embryos were analyzed at E10.5 after being sexed and genotyped. For somatic tissues used in this study, wild-type and *Smchd1*^−/−^ male brain and spleen tissues were obtained from dissection of adult mice from *Smchd1* heterozygote intercrosses. Cortex was obtained from a newborn F_1_ mouse at 3 days postpartum from a CAST × C57BL6 cross.

Prior to microarray data preprocessing, it was confirmed that the quality of all microarray data satisfied both the Affymetrix-recommended and CSC/IC Microarray Centre in-house quality control criteria. Processing of microarray data was performed using Bioconductor packages in R. Individual E10.5 embryos (wild type or mutant) were hybridized on individual arrays. For each array, 25-mer probe hybridization intensity data were preprocessed using the Robust Multichip Average (RMA) algorithm in the “affy” R package (14). The arrays were preprocessed together as they showed a similar data structure, judging from box plots of perfect-match probe hybridization intensities. The RMA algorithm was chosen as it provides more precise measurements for probes with lower expression values and eventually leads to higher sensitivity and specificity in detecting differential gene expression. These strengths of the RMA algorithm are especially important in the context of this study, as the changes in gene expression resulting from derepression of Xi alleles would be no more than 2-fold.

After preprocessing, probe set-based expression data were log_2_ transformed. For preprocessed probe sets, their fold changes in expression (still log_2_ transformed) were calculated by subtracting the mean for control samples from the mean for mutant samples. Detection of differential expression was carried out by using linear models and specifically the empirical Bayes methods (15) implemented in the R/Bioconductor package “limma.” Multiple-testing correction was carried out in order to control the false-discovery rate (FDR) using the methods of Benjamini and Hochberg (59) as implemented in the R/Bioconductor package “multtest.”

To map probe sets to the genome, Ensembl microarray probe mapping data (from Ensembl release 49, April 2007 Genebuild, NCBI mouse assembly m37, 23,493 genes) were used. Any probe set which was mapped to more than one gene was considered to be nonspecific and was discarded from analysis. In this project, 25,794 out of 45,037 probe sets (57.27%) were mapped to unique locations in the genome, corresponding to 15,539 genes (66.14%) in the Ensembl gene set.

Combining differential expression (at the probe set level) and probe set mapping data, two categories of differentially expressed probe sets were defined: (i) those which were mapped to unique locations in the genome and with FDRs of ≤10% (the more stringent category) and (ii) those which mapped to unique locations in the genome with FDRs of between 10% and 30%. Out of all probe sets showing an FDR of ≤30%, downregulated and upregulated are defined as those with “actual” (i.e., non-log-transformed) changes of expression <1 and >1, respectively (e.g., a probe set with an actual change of 1.25 is 25% upregulated in mutant samples relative to controls). Following the definition of upregulated probe sets, upregulated genes are defined as those represented by at least one upregulated probe set and no downregulated probe set at an FDR of ≤30%. The expression value of an upregulated gene was calculated as the average of those of all associated upregulated probe sets. Genes which were not associated with any probe sets with statistically significant changes were considered to be showing no change in expression.

### Testing of clustering and associations with genomic sequence features.

To test whether the differentially expressed genes were clustered, we calculated the median distance (117,090 bp) between the Ensembl genes corresponding to the Affymetrix probe sets of these genes and compared this with the median distances for 1,000 randomly selected sets of 64 Affymetrix probe sets/Ensembl genes, for which the mean of median distances was 10 times larger (1,232,096 bp; 95% confidence intervals, 1,228,900 to 1,260,244 bp). With the data apparently being normally distributed, we performed a *t* test, generating a *P* value of <0.0001.

In determining whether specific sequence features were associated with these clusters, we focused on annotated repetitive sequences using the RepeatMasker annotation from the UCSC Genome Browser (table: rmsk). We made the assumption that a sequence feature associated with susceptibility to Smchd1 effects would have to be present at least once in every cluster and then compared the frequency of each such feature with those in 1,000 randomly selected regions of identical size from the X chromosome. As the distributions were not normally distributed, significance was calculated using a Wilcoxon rank sum test. Most annotated repetitive elements showed no enrichment, but two subtypes of mouse SINE were significantly enriched, the B2Mm1a (*P* = 2.8 × 10^−6^) and ID4 (*P* = 7.3 × 10^−7^) elements.

### Gene expression analysis by qPCR.

Total RNA was isolated from individual embryos using TRIzol reagent (Invitrogen) according to the manufacturer's instructions. RNA was treated with Turbo DNA-free reagent (Ambion) to remove all DNA contamination. cDNA synthesis was primed from random hexamers (GE Healthcare) with Superscript III reverse transcriptase (Invitrogen). Quantitative PCR (qPCR) was performed with SYBR green PCR master mix (Bio-Rad) on a Chromo4 real-time PCR System (Bio-Rad). All primer sequences and PCR conditions are available upon request. The sequences of the primers used for Fig. 2E can be found in references 2 and 16. A melting curve test was performed for each experiment to ensure the specificity of amplification. Data were normalized to expression of several housekeeping genes (*β-actin*, *Idh2*, *Hmbs*, and *Gapdh*). A representative example is shown in the figures. Each amplicon was analyzed in duplicate at least three times on cDNA preparations from individual embryos.

### Bisulfite treatment and analysis by Sequenom or sequencing.

Genomic DNA was isolated from individual embryos and somatic tissues (brain or spleen) using standard methods. Bisulfite conversion of DNA was performed using the Epitect bisulfite kit (Qiagen) according to the manufacturer's instructions. All primer sequences and PCR conditions for amplifying from bisulfite-treated DNA are available upon request. The final PCR product in each case was loaded on a gel to ensure accurate amplification of a single band of correct size. For Sequenom analysis, all primers incorporate a T7 promoter tag and PCR products were analyzed by matrix-assisted laser desorption ionization–time of flight (MALDI-TOF) mass spectrometry after *in vitro* transcription and specific cleavage by Sequenom Inc. (17). For each amplicon, three independent DNA samples were analyzed, except for somatic tissues, where only one sample was analyzed. For conventional bisulfite analysis, PCR products were gel purified and subcloned into TOPO TA vector (Invitrogen). Individual colonies were analyzed by direct sequencing using the M13R universal primer.

### Accession number for microarray experiments and differential expression analysis.

Details of sample preparation and hybridization for microarray analysis have been deposited in ArrayExpress under experiment accession number E-MTAB-921.

## RESULTS

### Global gene expression analysis of *Smchd1*^−/−^ embryos.

**Fig 1:**
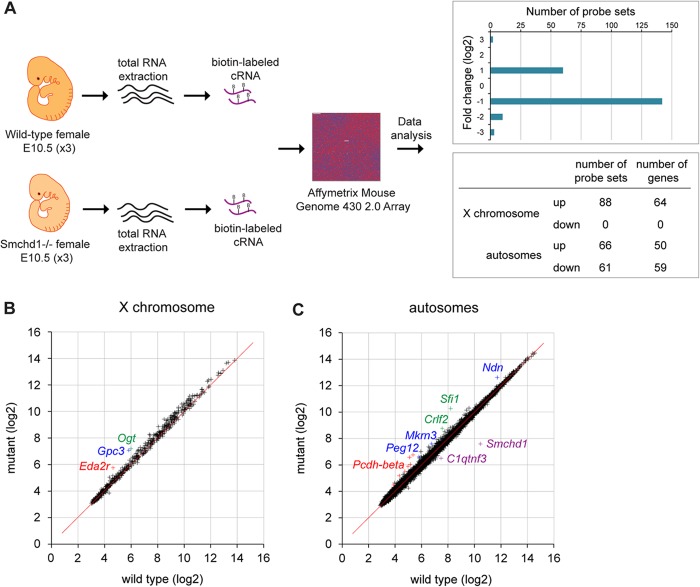
Comparative expression analysis of female wild-type and *Smchd1*^*−/−*^ embryos at E10.5. (A) Total RNAs from three female wild-type and three *Smchd1*^*−/−*^ embryos were processed individually and hybridized to Affymetrix mouse genome 430 2.0 arrays. A total of 215 probe sets were found to be differentially expressed following data processing (FDR, ≤30%), with 154 up- and 61 downregulated probe sets in *Smchd1*^*−/−*^ embryos showing a log_2_ fold expression change ranging from −3 to +3 and overlapping X-linked and autosomal genes. (B) Scatter plot of normalized expression values (in log_2_ scale) of 569 X-linked genes in wild-type versus mutant embryos. Three individual genes upregulated in mutant embryos are highlighted in color. (C) Scatter plot of normalized expression values (in log_2_ scale) of 14,962 autosomal genes in wild-type versus mutant embryos. A number of individual genes that are up- or downregulated in mutant embryos are highlighted in color.

The Smchd1 protein was previously shown to be important for gene silencing on the inactive X chromosome based on qPCR analysis of a small number of candidate genes in postimplantation embryos (2). In order to more fully understand the role of Smchd1 in Xi gene silencing and also to investigate if Smchd1 plays a role in regulating autosomal gene expression, we carried out a comparative microarray gene expression analysis of wild-type and *Smchd1*^−/−^ female embryos at E10.5, preceding female-specific embryonic lethality. The previously reported upregulation of X-linked genes in *Smchd1*^*−/−*^ embryos (2) provided a reference point for determining significant changes in gene expression. Total RNAs were extracted from three individual wild-type and *Smchd1*^*−/−*^ female embryos, and cRNAs prepared from these samples were hybridized to Affymetrix mouse genome 430 2.0 arrays (Fig. 1A). Upregulated and downregulated probe sets, i.e., those showing significant fold changes in expression between wild-type and *Smchd1*^*−/−*^ backgrounds, were then identified and mapped to Ensembl genes as described in Materials and Methods. Using a false-discovery rate of <10%, we uncovered a list of 63 differentially expressed probe sets, covering 17 autosomal genes and 30 genes on the X chromosome. A number of X-linked genes that were previously found to be upregulated in female mutant embryos by qPCR (2) were, however, absent from this list. Closer analysis revealed that probe sets for these genes did indicate upregulation but were scored with a low *P* value, suggesting that the initial cutoff was too stringent for genes that show a maximum 2-fold increase in expression, i.e., resulting from a shift from a monoallelic to a biallelic expression pattern in mutant embryos. With this in mind, we increased the false-discovery rate threshold to ≤30%, revealing an increased number of targets comprising 215 differentially expressed probe sets (see the supplemental material) covering 64 X-linked genes and 109 autosomal genes (Fig. 1A). All X-linked genes show upregulation, whereas autosomal genes are found to be either up- or downregulated.

### Clustering of Xi genes expressed in *Smchd1*^−/−^ female embryos.

**Fig 2:**
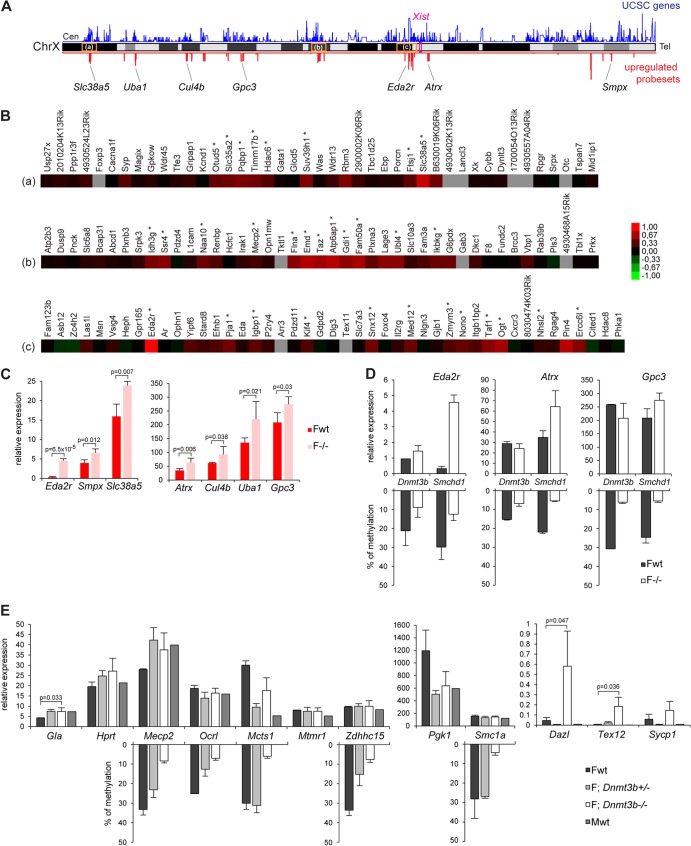
X-linked genes upregulated in mutant embryos are located in clusters, and upregulation can be uncoupled from DNA hypomethylation. (A) Chromosomal locations along the X chromosome of the 88 significantly upregulated probe sets on the array in female *Smchd1*^*−/−*^ embryos (red). Gene density (UCSC genes) along the chromosome is shown in blue. The positions of *Xist* and of the 7 X-linked genes analyzed in panel C are shown, as well as the locations of the three clusters highlighted in the heat maps in panel B (a, b, and c). Cen, centromere. Tel, telomere. (B) Heat maps of fold changes in expression between wild-type and mutant embryos for 3 gene clusters. (C) Expression levels of seven X-linked genes were measured by qPCR after reverse transcription in female (F) wild-type (wt) and *Smchd1* mutant (−/−) E10.5 embryos. (D) Expression levels of three X-linked genes were measured by RT-qPCR in wild-type (wt) and *Dnmt3b* mutant (−/−) E9.5 embryos and directly compared with data obtained for wild-type and *Smchd1*^*−/−*^ embryos from panel C. Methylation levels, shown as a reverse histogram, were measured for the *Eda2r*, *Atrx*, and *Gpc3* CGIs in wt and *Dnmt3b*^−/−^ E9.5 embryos and wt and *Smchd1*^*−/−*^ E10.5 embryos by Sequenom. (E) Expression levels of nine X-linked genes and three autosomal targets were measured by RT-qPCR in female (F) and male (M) wild-type (wt), *Dnmt3b*^*+/−*^, and *Dnmt3b*^*−/−*^ E9.5 embryos. Methylation levels, shown as a reverse histogram, were analyzed using bisulfite Sequenom analysis for five genes in female E9.5 wt and *Dnmt3b* heterozygous and homozygous mutant embryos. For all genes, graphs represent the mean from at least 3 individual embryos ± standard deviation of the mean (except for *Dnmt3b* E9.5 wt samples, where *n* = 2). For expression analysis, levels are normalized to housekeeping gene expression. When significant, *P* values for fold change in expression are indicated (*P* ≤ 0.05, unpaired Student *t* test).

More than 50% of genes (64 out of 114) showing significant upregulation in female mutant embryos are located on the X chromosome. These 64 genes (see the supplemental material) account for around 10% of X-linked genes represented on the array (*n* = 569), indicating, as we previously reported (2), that only a subset of genes undergo reactivation despite a global hypomethylation of the Xi (2, 4). Visualization of normalized X-linked gene expression levels between wild-type and mutant embryos by scatter plotting revealed that the data cloud deviates slightly from the diagonal (Fig. 1B), indicating that expression levels may be weakly increased in a more global manner for the chromosome but can be scored as significant for only a subset of genes. Significant overexpression found for the 64 genes ranges from 1.2 to 2 (see the supplemental material). We previously reported upregulation in mutant females for 6 genes tested at E9.5/E10.5 by qPCR (2). Four of these (*Hprt*, *Mcts1*, *Mecp2*, and *Smc1a*) are found in our list. The *Pgk1* gene is covered by one probe set showing increased expression in mutant, but as this maps to multiple locations in the genome, it was eliminated from the final list. Xi gene upregulation detected by microarray analysis was further validated by qPCR analysis for 7 randomly selected genes, *Eda2r*, *Smpx*, *Slc38a5*, *Atrx*, *Cul4b*, *Uba1*, and *Gpc3* (Fig. 2C). Additionally, as a control we randomly selected 6 genes that were not scored as derepressed (*Brwd3*, *Slc6a8*, *Sh3kbp1*, *Trmt2b*, *Rab33a*, and *Tmsb4x*) and observed similar expression levels in wild-type and mutant embryos (data not shown).

**Fig 3:**
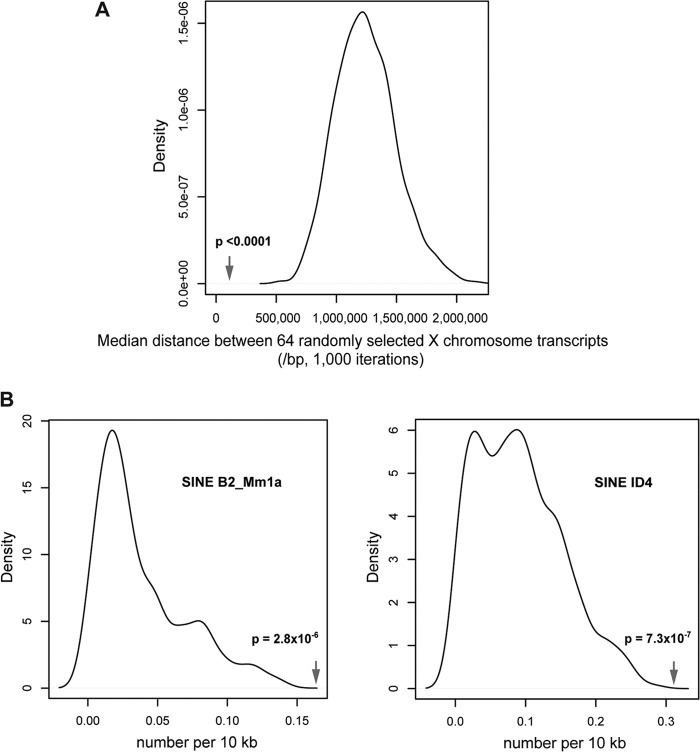
Clustering and genomic sequence feature analyses on the X chromosome. (A) Nonrandom clustering of the differentially expressed genes is illustrated by the median distance between the differentially expressed genes (arrow) compared with the increased median distances of 64 genes randomly selected 1,000 times from the mouse X chromosome. (B) Enrichment for two mouse SINE subtypes in the X-linked clusters is shown by their relative frequencies within the clusters defined by abnormal expression due to the loss of Smchd1, compared with the lower frequencies for the same elements in 1,000 randomly selected regions of equivalent size in the X chromosome.

Analysis of the chromosomal locations of the 88 X-linked probe sets significantly upregulated in female mutant embryos demonstrated that they are distributed across the entire chromosome (Fig. 2A). As previously observed, there is no correlation with gene position relative to the *Xist* locus (2). However, 70% of the probe sets were associated with genes within closely linked clusters (Fig. 2A) (see the supplemental material). The significant tendency to clustering was confirmed by showing a decreased median distance between these 64 genes compared with those of 1,000 randomly sampled sets of 64 genes from the X chromosome (*P* < 0.0001) (Fig. 3A). We could identify nine different regions where genes are clustered by groups ranging from 2 to 12 genes and from 65 kb to 1.5 Mb in size (see the supplemental material). Examples of three of these clusters and surrounding genes are shown as heat maps for the fold change in expression between wild-type and mutant embryos in Fig. 2B. However, it is important to note that not all genes located within the defined clusters show upregulation in mutant embryos. The clusters generally map to gene-rich regions on the X chromosome (Fig. 2A). We tested whether the observed clustering of genes is associated with genomic sequence features in the immediate chromosomal environment, such as density of any particular type of repetitive sequence or family of transposable elements, as defined by repeat masker annotations. We found a significant enrichment only with two types of repetitive elements around upregulated clusters, the B2_Mm1a and ID4 SINE subtypes (Fig. 3B). We also assessed whether derepressed gene clusters map to defined topology-associated domains (TADs), which are self-associating chromosomal domains defined by 5C and HiC interaction maps in which genes can be coordinately regulated (18, 19). Again, we did not find any clear correlation between the Smchd1-regulated gene clusters and defined TADs on Xi.

### CGI hypomethylation is not sufficient to account for Xi gene derepression in Smchd1 mutant embryos.

We previously showed that Smchd1 is required for methylation of a large proportion of Xi CGIs, and we have suggested that this could account for the observed Xi gene derepression (2, 4). Consistent with this, 95% of the genes upregulated in mutant embryos are hypomethylated in the absence of Smchd1 in female mouse embryo fibroblast (MEF) cells (4). To further test the proposed link between CGI hypomethylation and derepression of Xi genes in *Smchd1*^*−/−*^ embryos, we used qPCR to determine the expression levels of several X-linked genes in female embryos deficient in the *de novo* DNA methyltransferase Dnmt3b, which is required for CGI methylation on Xi (4) (Fig. 2D and E). Comparing expression levels with those for matched wild-type control embryos, we observed derepression of three genes (*Eda2r* [Fig. 2D] and *Gla* and *Mecp2* [Fig. 2E]) but not to the extent seen in *Smchd1*^*−/−*^ embryos (Fig. 2C) (2). Moreover, no significant effects were observed at a further six genes that are derepressed in *Smchd1*^−/−^ female embryos (*Atrx*, *Gpc3*, *Hprt*, *Mcts1*, *Pgk1*, and *Smc1a*) or at three other X-linked genes (*Ocrl*, *Mtmr1*, and *Zdhhc15*) (Fig. 2D and E). As a control, we analyzed three autosomal loci, *Dazl*, *Tex12*, and *Sycp1*, previously shown to be hypomethylated and derepressed in *Dnmt3b*^*−/−*^ embryos (16). In all cases, we observed derepression as previously reported (Fig. 2E).

Using Sequenom Epityper analysis, we confirmed Xi CGI hypomethylation in *Dnmt3b*^−/−^ embryos at eight of the loci analyzed, *Eda2r*, *Atrx*, and *Gpc3* (Fig. 2D) and *Mecp2*, *Ocrl*, *Mcts1*, *Zdhhc15*, and *Smc1a* (Fig. 2E). Hypomethylation of *Hprt* and *Pgk1* Xi CGIs in *Dnmt3b*^−/−^ embryos was shown previously (4). Collectively, these results suggest that the derepression of specific Xi genes observed in *Smchd1*^−/−^ embryos cannot be attributed exclusively to hypomethylation of their associated CGIs.

### Misexpression of autosomal loci in *Smchd1*^−/−^ embryos.

Although scatter plot visualization of normalized gene expression levels indicated that global expression of autosomal genes is relatively unaffected in *Smchd1*^−/−^ embryos (Fig. 1C), significant changes of expression were observed for 109 genes. Gene ontology analysis did not show overrepresentation for any particular term (data not shown). Fifty-nine of 109 autosomal genes are downregulated in mutant embryos, with the *Smchd1* gene showing the highest level of downregulation, as seen on the scatter plot in Fig. 1C. Reduction in *Smchd1* transcript expression is likely to be due to nonsense-mediated mRNA decay, as previously suggested (2). Other downregulated genes show only slight decreases in expression in mutant embryos. The remaining 50 autosomal genes show significant upregulation in mutant embryos (see the supplemental material), with the majority showing a degree of overexpression ranging from 1.2 to 1.8 and a few higher than 2. Interestingly, two clusters of autosomal genes subject to monoallelic expression were upregulated in *Smchd1*^−/−^ embryos (see the supplemental material). The first comprises four genes from the region homologous to the PWS region on chromosome 7C, which are subject to parental imprinting (10). The second encompasses several genes from the *Pcdha* and *Pcdhb* gene clusters on chromosome 18. The *Pcdha* gene cluster is expressed in a random monoallelic fashion in single neurons in the mouse (11).

**Fig 4:**
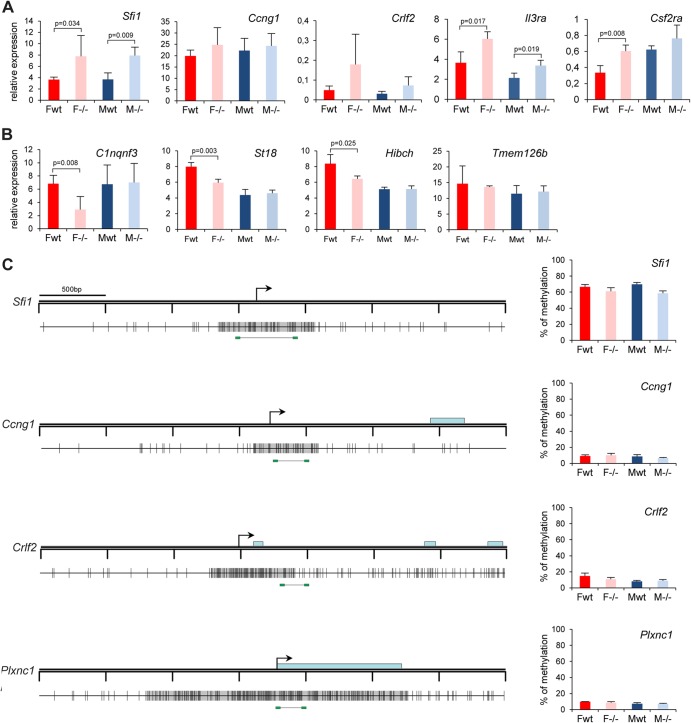
Expression and DNA methylation analysis of candidate targets identified in the array experiment. Expression levels of five upregulated (A) and four downregulated (B) candidate genes were assessed by RT-qPCR in female (F) and male (M) wild-type (wt) and *Smchd1* mutant (−/−) embryos at E10.5. For all genes, graphs represent the mean from at least 3 individual embryos ± standard deviation of the mean. Expression levels are normalized to housekeeping gene expression. When significant, *P* values for fold change in expression are indicated (*P* ≤ 0.05, unpaired Student *t* test). (C) Methylation levels of the CGIs linked to four candidate genes (all upregulated in the microarray experiment) were measured using bisulfite Sequenom analysis. A schematic map of a 3.5-kb genomic region around the transcription start site (arrows) with CGIs (gray boxes) for each gene is shown on the left. Blue rectangles represent coding sequences. Vertical bars indicate the positions of CpG sites, and the line linking by two green rectangles at the bottom indicates the position of the fragment analyzed by Sequenom. Graphs on the right indicate methylation levels calculated from an average of 3 independent embryos from each sex and each genotype. Error bars represent standard deviations.

Misexpression of autosomal genes could theoretically be a secondary consequence of Xi gene derepression, and consistent with this, qPCR analysis confirmed downregulation of *C1qtnf3*, *St18*, and *Hibch* in female but not in male mutant embryos (Fig. 4B). However, other misexpressed genes, for example, the upregulated genes *SfiI*, *Crlf2*, *Il3ra*, and *Csf2ra* (Fig. 4A) and the PWS and *Pcdha* gene clusters (see below), were overexpressed in both male and female mutant embryos, indicating most likely a direct role for Smchd1 in regulating these loci. Interestingly, three of these genes (*Il3ra*, *Csf2ra*, and *Crlf2*) are located on the pseudoautosomal region on the human X chromosome and have translocated to autosomes in the mouse. Unlike the case for genes on Xi, the increased expression of *SfiI* and *Crlf2* was not correlated with a reduction in DNA methylation at promoter regions (Fig. 4C), although reduced DNA methylation was observed at both the PWS and *Pcdha* gene clusters (see below).

### Loss of imprinting at the PWS gene cluster in *Smchd1*^−/−^ embryos and adult tissues.

**Fig 5:**
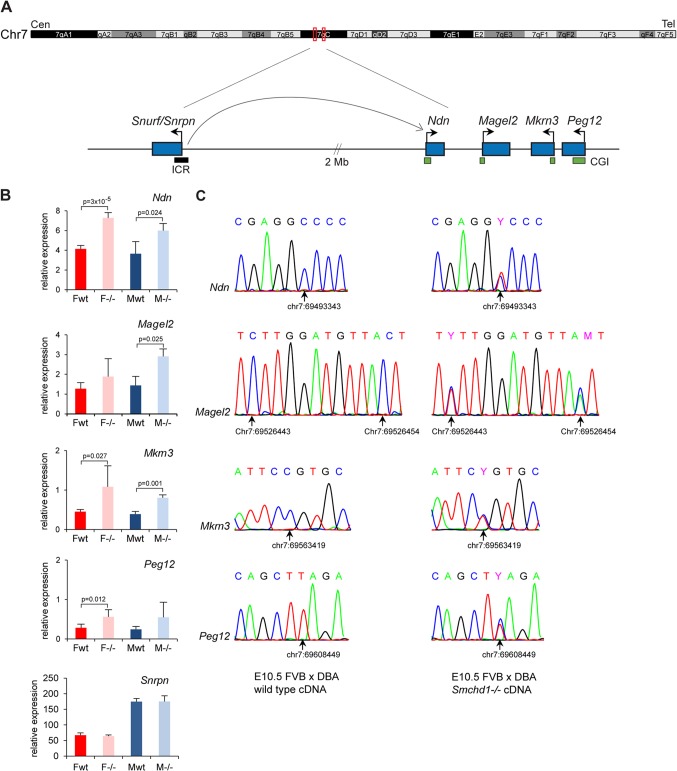
Imprinted genes from the PWS region are reactivated on the maternal allele in *Smchd1*^*−/−*^ embryos. (A) Schematic diagram of the PWS region located on chromosome 7C in the mouse. The imprinted genes analyzed cluster in a region of 116 kb, while *Snurf*/*Snprn*, which comprises the imprinting control region (ICR), is located 2 Mb upstream. Arrows indicate the direction of transcription of each gene. CpG islands (CGIs) are shown as green boxes, and the ICR is shown as a black box. Cen, centromere. Tel, telomere. (B) Expression levels of the four clustered PWS genes *Ndn*, *Magel2*, *Mkrn3*, and *Peg12*, plus *Snrpn*, were measured by RT-qPCR in female (F) and male (M) wild-type (wt) and mutant (−/−) embryos at E10.5. For all genes, graphs represent the mean from at least 3 individual embryos ± standard deviation of the mean. Expression levels are normalized to housekeeping gene expression. When significant, *P* values for fold change in expression are indicated (*P* ≤ 0.05, unpaired Student *t* test). (C) Conventional sequencing analysis of RT-PCR products centered on FVB/DBA SNPs found in the four clustered genes was performed for wild-type and *Smchd1*^*−/−*^ embryos at E10.5. Chromatograms are shown for each gene, with SNP position (arrow) and coordinate underneath, and indicate that the DBA paternal allele is expressed only in the wild type, whereas both alleles are expressed in *Smchd1*^*−/−*^ embryos. The same results were obtained for 3 individual embryos from each sex and genotype.

The PWS region, depicted in Fig. 5A, is located on chromosome 7C in the mouse and comprises a number of paternally expressed genes. The imprinted status of the PWS region is controlled by a complex imprinting control region (ICR) located at the 5′ end of *Snurf-Snrpn* 2 Mb upstream (Fig. 5A) (20). The ICR also controls imprinted genes located proximally (21, 22). We characterized in detail the expression pattern in embryos of genes from the PWS region. The four genes found in our gene list (*Ndn*, *Magel2*, *Mkrn3*, and *Peg12*), all paternally expressed, were tested by reverse transcription-qPCR (RT-qPCR). A 2-fold upregulation was observed for all four genes in E10.5 *Smchd1*^*−/−*^ embryos (Fig. 5B), suggesting that the silent maternal allele might be reactivated. Equivalent upregulation was observed in both female and male mutant embryos, demonstrating that the observed effect is not a secondary consequence of misregulating genes on Xi.

To test for loss of imprinting, we identified single nucleotide polymorphisms (SNPs) in the coding regions of the 4 genes between FVB/N and DBA/2J mouse strains. The presence of SNPs was confirmed by PCR and sequencing on genomic DNA from both mouse strains. *Smchd1*^*+/−*^ heterozygote mice were backcrossed twice to the DBA/2J strain. Male F_2_ progeny homozygous for DBA/2J SNPs and heterozygous for the *Smchd1* mutation were crossed with FVB/N *Smchd1* heterozygote females, and embryos were analyzed at E10.5 by RT-PCR followed by sequencing. The results, shown in Fig. 5C, demonstrate that both paternal and maternal alleles are expressed at similar levels in *Smchd1*^*−/−*^ embryos, contrasting with the case for wild-type embryos, in which all of the genes are expressed solely from the paternal allele. *Snrpn* expression levels were also analyzed and found to remain unchanged in *Smchd1*^*−/−*^ embryos (Fig. 5B).

**Fig 6:**
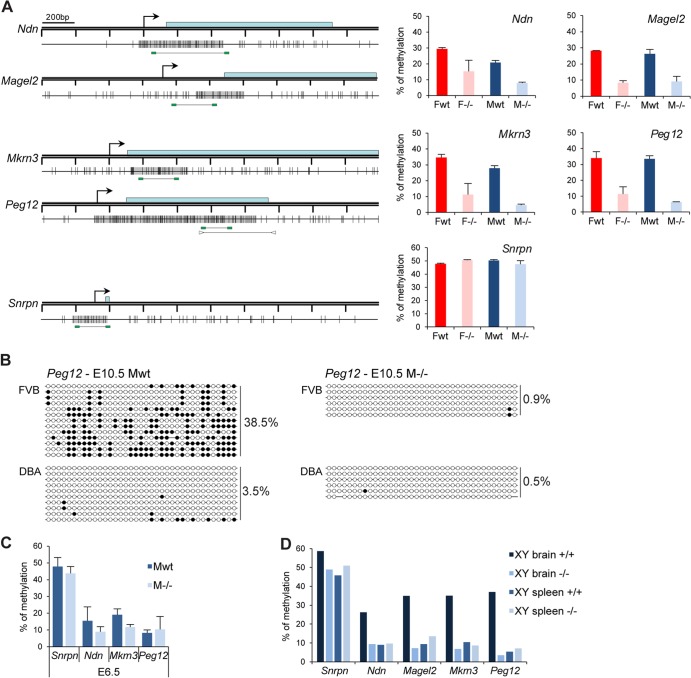
The clustered PWS genes are hypomethylated in *Smchd1*^*−/−*^ embryos. (A) Methylation analysis by Sequenom of fragments encompassing the CGIs linked to genes from the PWS cluster. A schematic map of a 2-kb genomic region around the transcription start site (arrows) with CpG islands (gray boxes) for each gene is shown on the left. Blue rectangles represent coding sequences. Vertical bars indicate the positions of CpG sites, and the line linking two green rectangles at the bottom indicates the position of the fragment analyzed by Sequenom. For *Peg12*, arrowheads represent primers used for conventional bisulfite in panel B. Graphs on the right indicate methylation levels calculated from an average of three E10.5 embryos from each sex (F, female; M, male) and genotype (wt, wild type; −/−, *Smchd1* mutant). Error bars represent standard deviations. (B) Conventional bisulfite analysis of the *Peg12* CGI in wild-type and *Smchd1*^*−/−*^ male FVB/DBA embryos at E10.5. Circles represent CpG sites either unmethylated (open) or methylated (closed), and global methylation levels are indicated on the right for each allele and each sample analyzed. (C) Average methylation levels measured by bisulfite analysis using Sequenom from 3 individual male wild-type and −/− embryos at E6.5. Error bars represent standard deviations. (D) Methylation levels measured in brain and spleen tissues from wild-type and *Smchd1*^*−/−*^ adult males (XY).

Next, given the link between Smchd1 function and CGI methylation on the inactive X chromosome, we set out to determine whether or not Smchd1 is required for DNA methylation at these autosomal imprinted loci. We examined the DNA methylation patterns of CGIs associated with the four PWS genes in E10.5 embryos using Sequenom bisulfite analysis. Methylation levels were 30 to 40% in wild-type embryos (Fig. 6A), consistent with a uniparental pattern of methylation characteristic of imprinted gene differentially methylated regions (DMRs) (23). This uniparental pattern was confirmed with SNPs for *Peg12* in (FVB × DBA)F_1_ embryos (Fig. 6B) and for *Magel2* in the cortex of a (Cast × B6)F_1_ neonate mouse (data not shown), using conventional bisulfite sequencing. However, in *Smchd1*^*−/−*^ male and female embryos, all DMRs were found to be significantly hypomethylated compared to those in wild-type controls (Fig. 6A). To confirm this, we determined the methylation profile of the *Peg12* CGI in (FVB × DBA)F_1_ *Smchd1*^*−/−*^ E10.5 embryos by bisulfite sequencing. The results, shown in Fig. 6B, indicate that the methylated maternal allele is entirely hypomethylated in *Smchd1*^*−/−*^ embryos. *Snprn* methylation levels were around 50% in *Smchd1*^*−/−*^ embryos, as seen in the wild type (Fig. 6A), suggesting that Smchd1 does not affect germ line imprint maintenance at *Snrpn*.

Analysis of CGI methylation of male embryos at an earlier developmental stage (E6.5) indicated that levels were lower than at E10.5 for all PWS genes tested, consistent with the observation that methylation of these DMRs is established postimplantation (24, 25) (Fig. 6C). Differences between wild-type and *Smchd1*^*−/−*^ levels were not significant at this earlier stage, suggesting that Smchd1 may be critical for establishment of methylation at the DMRs analyzed. *Snrpn* methylation levels, established in the germ line, were, in contrast, comparable to E10.5 levels (Fig. 6C).

*Ndn* and *Magel2* genes are expressed predominantly in the brain. We measured methylation levels of *Ndn*, *Magel2*, *Mkrn3*, and *Peg12* DMRs in brain and spleen tissues from wild-type and *Smchd1*^*−/−*^ adult males. The data shown in Fig. 6D indicate that PWS imprinted genes are methylated in adult brain but not in spleen. In *Smchd1*^*−/−*^ adult males the DMRs were hypomethylated, consistent with data obtained for embryos. In summary, these results demonstrate a requirement for Smchd1 in CGI methylation and silencing of PWS cluster genes.

### Smchd1 is not required for DMR methylation at the majority of imprinted loci.

**Fig 7:**
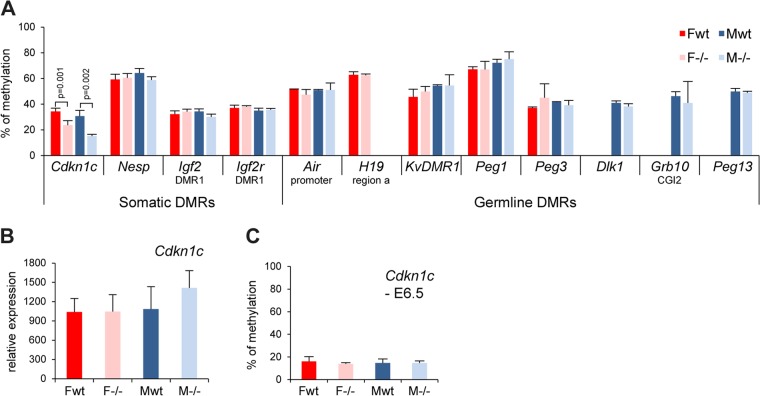
Germ line and somatic DMR methylation is generally unaffected in *Smchd1*^−/−^ embryos. (A) Methylation levels analysis using Sequenom for a number of germ line and somatic differentially methylated regions (DMRs) in female (F) and male (M) wt and −/− E10.5 embryos. Significant *P* values are indicated for the *Cdkn1c* DMR (*P* ≤ 0.05, unpaired Student *t* test). (B) Expression levels of the *Cdkn1c* gene in female and male wt and −/− E10.5 embryos. (C) Analysis of the methylation levels of the *Cdkn1c* somatic DMR at E6.5. Graphs represent an average from 3 independent embryos at E10.5 and E6.5 ± standard deviation. For expression analysis, levels are normalized to housekeeping gene expression.

In light of our observations on the role of Smchd1 in maintaining normal methylation levels at the PWS imprinted genes, we asked whether Smchd1 could influence DMR methylation at other imprinted loci. We examined the methylation levels of a number of known primary (or germ line) DMRs, which acquire methylation in the germ line during gametogenesis, and secondary (or somatic) DMRs, for which allelic methylation patterns are established postfertilization, most probably through the influence of primary DMRs (26). We used Sequenom bisulfite analysis in E10.5 embryos. Eight germ line DMRs (*Air*, *H19*, *KvDMR1*, *Peg1*, *Peg3*, *Dlk1*, *Grb10*, and *Peg13*) were analyzed and did not show any differences between wild-type and *Smchd1*^*−/−*^ E10.5 embryos in both males and females (Fig. 7A). Four somatic DMRs that acquire DNA methylation at various stages postimplantation (*Cdkn1c*, *Nesp*, *Igf2r*-DMR1, and *Igf2*-DMR1) were analyzed. A significant reduction of methylation was observed in both male and female mutant embryos at E10.5 for the 5′ DMR of the *Cdkn1c* gene only (Fig. 7A), although this did not correlate with any significant change in gene expression (Fig. 7B). Analysis of the *Cdkn1c* DMR at E6.5 revealed that methylation levels are low at this stage, and there was no detectable difference between wild-type and *Smchd1*^*−/−*^ embryos (Fig. 7C). Thus, Smchd1 contributes to postimplantation establishment of methylation at *Cdkn1c* but not at other imprinted loci that were tested.

### Misregulation of the *Pcdha* and *Pcdhb* gene clusters in *Smchd1*^*−/−*^ embryos and adults.

**Fig 8:**
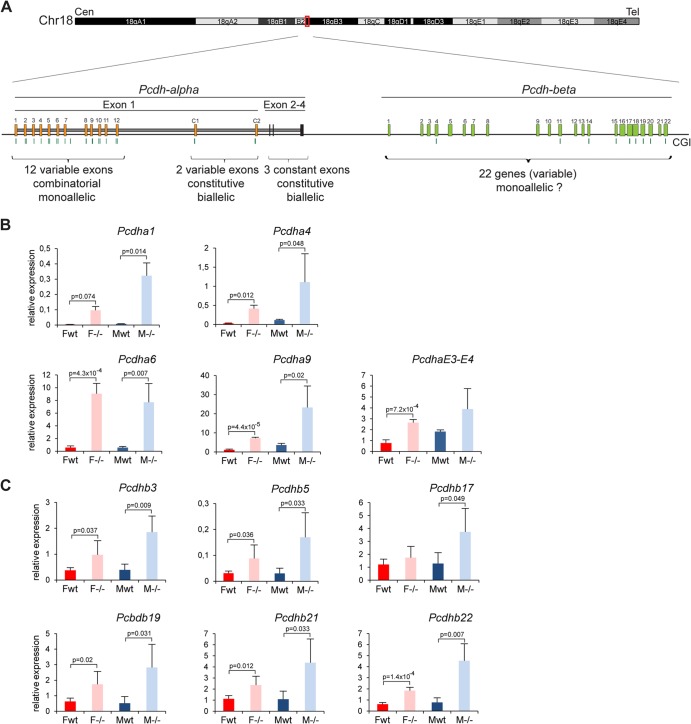
Genes from the *Pcdh-alpha* and *Pcdh-beta* clusters are upregulated in *Smchd1*^*−/−*^ embryos. (A) Schematic diagram of the *Pcdha* and *Pcdhb* gene clusters covering a region of 600 kb on chromosome 18 in the mouse. The *Pcdha* cluster is composed of 12 variable-region exons (1 to 12) showing combinatorial and monoallelic expression, 2 variable-region exons (C1 and C2), and 3 constant-region exons (E2 to -4), constitutively and biallelically expressed in single neurons. The *Pcdhb* cluster is composed of 22 individual genes, for which the allelic pattern of expression in single cells is not known. Cen, centromere. Tel, telomere. (B and C) Expression levels of several *Pcdha* exons (B) and *Pcdhb* genes (C) were measured by RT-qPCR in female (F) and male (M) wild-type (wt) and mutant (−/−) embryos at E10.5. For all genes, graphs represent the mean from 3 or 4 individual embryos ± standard deviation of the mean. Expression levels are normalized to housekeeping gene expression. When significant, *P* values for expression fold change are indicated (*P* ≤ 0.05, unpaired Student *t* test).

We next investigated the *Pcdh* cluster of genes on chromosome 18. The *Pcdh* locus is tandemly organized in three clusters defined by three *Pcdh* subfamilies, *Pcdha*, *Pcdhb*, and *Protocadherin-gamma* (*Pcdhg*), each of which has a specific genomic organization (Fig. 8A) (27). The *Pcdha* and *Pcdhg* clusters are comprised of multiple variable exons with autonomous promoters and a set of constant exons to which each variable transcript is spliced. The *Pcdhb* cluster is defined by a number of independent transcription units that are sequentially organized (Fig. 8A). Single-cell analysis of cells from the cerebellar cortex showed that *Pcdha* and the *Pcdhg* genes exhibit monoallelic and combinatorial expression of exons in individual Purkinje neurons (11, 28). *Pcdhb* genes were not tested, and it is therefore unknown whether they are expressed monoallelically or biallelically.

Several probe sets overlapping the *Pcdha* and *Pcdhb* gene clusters were found to be highly upregulated in our microarray experiment (see the supplemental material). Quantitative RT-PCR analysis confirmed the microarray data, showing a 2- to 10-fold upregulation of all *Pcdha* and *Pcdhb* genes tested, including genes for which overlapping probe sets did not show upregulation in the microarray analysis (Fig. 8B and C). This may indicate that *Pcdha* variable exons are expressed biallelically in the *Smchd1*^*−/−*^ background, unlike in the wild type, and/or it could indicate that these genes, which are normally expressed only in restricted neuronal cell types, become expressed in a more ubiquitous manner in *Smchd1*^*−/−*^ embryos. Again, upregulation occurs in both female and male *Smchd1*^*−/−*^ E10.5 embryos (Fig. 8B and C), indicating that Xi-related indirect effects do not play a role.

**Fig 9:**
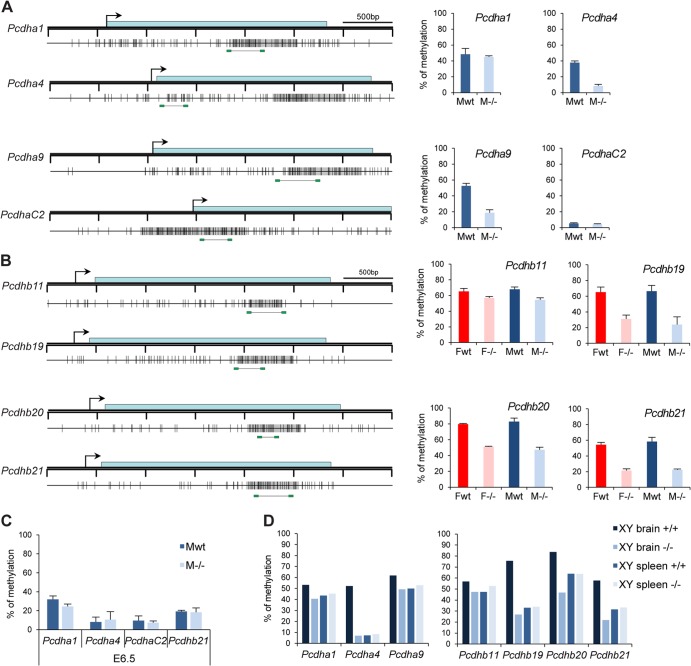
Hypomethylation of *Pcdh*-linked CGI in *Smchd1*^*−/−*^ embryos. (A and B) Methylation analysis by Sequenom of fragments encompassing the CGIs linked to genes from the *Pcdha* (A) and *Pcdhb* (B) cluster. A schematic map of a 3.5-kb genomic region around the transcription start site (arrows) with CGIs (gray boxes) for each gene is shown on the left. Blue rectangles represent coding sequences. Vertical bars indicate the positions of CpG sites, and the line linking two green rectangles at the bottom indicates the position of the fragment analyzed by Sequenom. Graphs on the right indicate methylation levels calculated from an average of 3 independent E10.5 embryos from each sex (F, female; M, male) and genotype (wt, wild type; −/−, mutant). Error bars represent standard deviations. (C) Average methylation levels measured by bisulfite analysis using Sequenom from 3 individual male wild-type and −/− embryos at E6.5. Error bars represent standard deviations. (D) Methylation levels measured in brain and spleen tissues from wild-type and *Smchd1*^*−/−*^ adult males (XY).

We went on to analyze the methylation profile of the region. All *Pcdh* genes display a high density of CpG dinucleotides (29). These CpG-dense regions are located across the body of *Pcdha* variable exons and *Pcdhb* genes, and most are annotated as CGIs. Previous studies indicated that CGIs within this region are densely methylated (30). The methylation patterns of the CGIs linked to several *Pcdha* variable exons and *Pcdhb* genes were assessed using Sequenom bisulfite analysis of male and female E10.5 embryos. The results obtained showed 40 to 80% methylation in wild-type E10.5 embryos and in several cases significant hypomethylation in *Smchd1*^*−/−*^ embryos (Fig. 9A and B), suggesting that Smchd1 plays a role in establishing or maintaining normal methylation levels in this region. These findings were substantiated using conventional bisulfite sequencing of selected loci (data not shown). This analysis also demonstrated that methylation of *Pcdha* and *Pcdhb* CGIs occurs equally on both parental alleles. These observations do not rule out random allele-specific methylation restricted to the limited population of Purkinje neurons, in which individual *Pcdh* genes are expressed.

Methylation analysis of selected *Pcdh* CGIs at E6.5 revealed that methylation levels are low and, moreover, that there is no difference between wild-type and *Smchd1*^*−/−*^ genotypes at this earlier developmental stage (Fig. 9C). This is highly reminiscent of our observations for the PWS cluster and *Cdkn1c* (Fig. 6C and 7C) and indicates that Smchd1 plays a role in establishing methylation patterns at selected neuronally expressed loci during postimplantation development.

Finally, we analyzed the methylation levels of *Pcdh* CGI in brain and spleen tissue from wild-type and mutant adult males (Fig. 9D). This revealed that *Pcdh* CGIs for which methylation is affected in *Smchd1*^*−/−*^ embryos (*Pcdhb19* and *Pcdhb21*) are subject to brain-specific methylation, which is then lost in *Smchd1*^*−/−*^ mutants. It is striking that, similar to the case for the PWS cluster, affected *Pcdh* loci are those that normally acquire methylation during development of neuronal but not other (spleen) lineages.

## DISCUSSION

### The function of Smchd1 on the inactive X chromosome.

In this study, we showed that the widespread hypomethylation of CGIs observed in *Smchd1*^*−/−*^ female embryos is accompanied by loss of silencing of only a subset of X-linked genes (around 10%). Interestingly, a large proportion of these genes are located in clusters, and we found a correlation with a significant enrichment of only two SINE subfamilies. In future studies, to investigate what could account for derepressed gene clusters, it will be interesting to analyze the epigenomic environment and determine if specific chromatin signatures, for example, histone modifications or histone variants, define the propensity for Smchd1 dependence. Additionally, given the link between transcriptional activity and replication timing in XCI (31), it will be interesting to investigate whether Smchd1 dependence relates to replication timing domains on Xi.

By comparing changes in expression levels of X-linked genes between female *Dnmt3b*^*−/−*^ and *Smchd1*^−/−^ embryos, we found that CGI hypomethylation is not sufficient to account for Smchd1-linked gene derepression. The analysis of *Dnmt3b*^*−/−*^ embryos is in line with data obtained for human cells deficient in Dnmt3b (32). Taken together, these observations indicate that Smchd1 likely controls other silencing pathways acting to keep the X chromosome fully silent. A possibility is that Smchd1 controls higher-order compaction of chromatin of the Xi, as has been reported for the related MORC ATPases in Arabidopsis (7) or for the human orthologous protein (33). Interestingly, it was recently shown that depletion of SMCHD1 from human somatic cells results in Xi decompaction (33). Alternatively, Smchd1 could mediate long-range interactions, a role attributed to the related cohesin complex SMC proteins, in the context of gene regulation (34, 35). The observed CGI hypomethylation of the Xi could therefore be an indirect effect caused by the absence of Smchd1. To test this scenario, it could be interesting to analyze whether Xi-linked genes are similarly upregulated in female *Smchd1*^*−/−*^ extraembryonic tissues, where DNA methylation is thought to play a relatively minor role in X inactivation (36).

### A role for Smchd1 in imprinting at the PWS cluster.

Analysis of gene expression in *Smchd1*^*−/−*^ E10.5 embryos identified loss of imprinting and biallelic expression of four clustered genes (*Ndn*, *Magel2*, *Mkrn3*, and *Peg12*) in the PWS region. One possible mechanism for this would be inappropriate activation of the maternal *Snurf/Snrpn* ICR, as previous studies have identified a role for the *Snurf/Snrpn* ICR in paternal activation of imprinted genes in the PWS region. Thus, paternal transmission of 4.8-kb deletion of the *Snprn* ICR results in partial loss of expression of *Ndn* and *Mkrn3* (37). In another study, a 42-kb deletion including the *Snrpn* gene was reported to result in a complete imprinting defect and silencing of *Ndn* and *Mkrn3* (20), indicating that the deleted region is necessary for paternal expression of *Ndn* and *Mkrn3* and most likely *Magel2* and *Peg12*, which lie more distally in the region. However, we did not observe hypomethylation of the maternal *Snurf/Snrpn* ICR that in other studies, for example, of mice carrying a maternally transmitted disruption of the *Dnmt3L* gene (38), resulted in biallelic expression of *Ndn* and *Mkrn3* (also known as *Zfp127*). Thus, our observations suggest that hypomethylation of the PWS cluster in *Smchd1*^*−/−*^ embryos is not attributable to disruption of ICR function. Consistent with this, we did not observe any misregulation of imprinted genes located proximally which are also controlled by the Snrpn ICR. Instead, we favor the idea that a constitutive Smchd1-dependent pathway governs default repression and DNA methylation of the *Ndn*, *Magel2*, *Mkrn3*, and *Peg12* CGIs in neuronal lineages and that activity of the unmethylated ICR on the paternal allele bypasses this pathway in *cis*, leading to neuronal expression of PWS cluster genes.

CpG methylation of DMRs associated with other imprinted loci was generally unaffected in *Smchd1*^*−/−*^ embryos, with the exception of a partial hypomethylation of the CGI associated with the *Cdkn1c* gene. It is possible that *Cdkn1c* is similarly subject to a constitutive pathway determining DNA methylation on the maternal allele. In this regard, it is interesting to note that mice deficient for the putative chromatin-remodelling factor Lsh show hypomethylation of *Cdkn1c* and also the PWS cluster genes but not of a number of other imprinted loci that were analyzed (39, 40).

### Smchd1 is required for epigenetic regulation of *Pcdha* and *Pcdhb* gene clusters.

Upregulation of a subset of *Pcdha* exons and *Pcdhb* genes in *Smchd1*^*−/−*^ embryos was detected using microarray analysis, and subsequent analysis by qPCR indicated that most if not all genes in these clusters are affected. *Pcdh* genes normally show highly tissue-specific expression, being restricted to specialized neurons (41), and levels of expression are generally very low in E10.5 embryos, close to the detection threshold on the microarray. The upregulation observed, together with the hypomethylation pattern at a number of the regions tested, suggests that the entire domain displays a more open and relaxed chromatin structure, permitting inappropriate expression. *Pcdha1* to *-a12* exons were shown to be monoallelically expressed in a random fashion in single neurons of the cerebellum (11), and it is tempting to speculate that the upregulation observed in *Smchd1*^*−/−*^ mutants may correspond to loss of silencing at the normally nonexpressed allele, although this remains to be tested. Single neurons usually express one or two *Pcdha* transcription units, monoallelically (11). Whether several genes could be expressed in single neurons (in a mono- or biallelic fashion) or whether the two alleles of a normally monoallelically expressed gene are active in a given neuron remains an open and interesting question. In future studies, this could be addressed using methods for single-cell analysis (42, 43). Our data concerning expression of *Pcdhb* genes suggest that this cluster is regulated similarly to the *Pcdha* cluster, although at present there is no direct evidence for monoallelic expression of *Pcdhb* genes.

Very little is known regarding the epigenetic control of expression of the *Pcdh* cluster or the *trans*-acting factors required for maintaining tissue-specific expression patterns. Our data and also an earlier study (44) suggest that DNA methylation is important for the regulation of *Pcdha* and *Pcdhb* clusters. The *Pcdh*-associated CGIs that we analyzed do not show uniparental methylation patterns, as is observed at DMRs of imprinted genes. This is consistent with the fact that each *Pcdh* transcription unit is expressed in only a small subset of neurons. Moreover, our data suggest that methylation of at least some *Pcdh*-linked CGIs occurs just before midgestation, at the time of specification of the neuronal lineages, and in a lineage-specific manner. Thus, a possible mechanism, drawing on that proposed above for the PWS cluster, is that *Pcdha* and *Pcdhb* clusters acquire CGI methylation through a constitutive Smchd1-dependent mechanism that normally functions specifically in neuronal lineages. Further extrapolating this comparison, we propose that activation of *Pcdha* and *Pcdhb* genes in individual Purkinje cell neurons could involve a single *cis*-acting regulatory element that bypasses the default pathway and protects against silencing/methylation at individual transcription units.

Several recent reports have demonstrated that CCCTC binding factor (CTCF) and the cohesin subunit Rad21 bind to transcriptionally active *Pcdha* promoters and enhancers, thereby mediating chromosomal interactions between promoters and enhancers and ensuring proper promoter choice at the *Pcdha* locus (45). In cells depleted of CTCF, expression of all *Pcdha* alternative exons tested declines markedly (46–48). In contrast, we observe an increase in expression levels of all genes tested. As DNA methylation prevents CTCF binding at these sites (45), the loss of methylation observed at *Pcdha* exons may render normally unavailable CTCF/cohesin binding sites accessible, resulting in miscontrolled expression of genes that are normally repressed.

It should be noted that at this point we cannot exclude the possibility that Smchd1 plays an indirect role in the silencing of the *PWS* and *Pcdh* loci by controlling the expression of a factor involved in the regulation of these two clusters.

### Physiological roles of Smchd1.

In light of our data demonstrating significant misexpression of both PWS and *Pcdha*/*Pcdhb* gene clusters, it is perhaps surprising that *Smchd1*^*−/−*^ male mice are viable and fertile. *Ndn* disruption results in behavioral alterations reminiscent of the human Prader-Willi syndrome (49, 50). However, the consequences of a biallelic expression of the *Ndn* gene and its three neighboring genes (*Magel2*, *Mkrn3*, and *Peg12*), especially in the brain, have not been documented. Protocadherins are cell adhesion molecules highly enriched in synaptic junctions and axons (27). Disruption of some *Pcdha* or *Pcdhg* sequences in mice causes defects in axonal projection of neurons (51) or impairment of synaptic formation and loss of neurons (52, 53). Again, the consequences of aberrant and biallelic expression of *Pcdh* genes are not known. With this in mind, it will be interesting in future studies to determine if *Smchd1*^*−/−*^ males exhibit behavioral or other neuronal-associated phenotypes.

Taken together, our data suggest that, in a physiological context, Smchd1 plays a central role in the establishment of DNA methylation and gene silencing on the inactive X chromosome and at a limited number of sites on autosomes, the PWS cluster, and the *Pcdh-alpha* and *-beta* gene clusters. It is not clear at present what links these different loci, although clearly they all represent examples of monoallelic expression. It is interesting to note that all affected loci lie in chromosomal environments with a high density of LINE-1 (L1) repeat elements, which have been implicated in gene silencing pathways (54–57). Somewhat paradoxically, we find that X-linked gene clusters upregulated in XX *Smchd1*^*−/−*^ embryos are associated with B2 SINE elements. A further link with specific repeat sequences has emerged with the identification of SMCHD1 mutations in FSHD1 families in which loss of epigenetic silencing at the D4Z4 macrosatellite repeat results in inappropriate derepression of the germ cell-specific DUX4 gene in skeletal muscle (58). Further studies will be necessary to test whether Smchd1 has a role in establishing heterochromatin at specific repeat sequence classes.

## Supplementary Material

Supplemental material
