# Supplementary material for: Epigenetic Functions of Smchd1 Repress Gene Clusters on the Inactive X Chromosome and on Autosomes
Source: Mol Cell Biol. 2013 Aug;33(16):3150–65. doi: 10.1128/MCB.00145-13 (PMC3753908; doi:10.1128/MCB.00145-13)
Supplement: Supplemental material [file supp_33_16_3150__index.html]

Supplemental material 

# Epigenetic Functions of Smchd1 Repress Gene Clusters on the Inactive X Chromosome and on Autosomes

## Supplemental material

**Files in this Data Supplement:**

- Supplemental file 1 -

  Dataset 1 (Genes upregulated and downregulated in Smchd1 mutant embryos)

  XLSX, 55K
